# Traditional and cyberbullying victimization as correlates of psychosocial distress and barriers to a healthy lifestyle among severely obese adolescents – a matched case–control study on prevalence and results from a cross-sectional study

**DOI:** 10.1186/1471-2458-14-224

**Published:** 2014-03-05

**Authors:** Ann DeSmet, Benedicte Deforche, Anne Hublet, Ann Tanghe, Evi Stremersch, Ilse De Bourdeaudhuij

**Affiliations:** 1 Department of Movement and Sport Sciences, Ghent University, Watersportlaan 2, B-9000 Ghent, Belgium; 2 Department of Public Health, Ghent University, Ghent, Belgium; 3 MPC Zeepreventorium, De Haan, Belgium

**Keywords:** Adolescence, Obesity, Cyberbullying, Peer victimization, Psychosocial functioning, Quality-of-life, Suicide, Healthy lifestyle, Physical activity

## Abstract

**Background:**

Obese youth are at increased risk for peer victimization, which may heighten their risk of psychosocial problems and physical activity avoidance, and lower the effectiveness of professional and lifestyle weight-loss initiatives. Little is known about obese adolescents’ risk for victimization from cyber-bullying and how this relates to psychosocial functioning and healthy lifestyle barriers. The purpose of the study was to assess traditional and cyber-victimization among adolescents with severe obesity and its relation to psychosocial distress and barriers to healthy lifestyles.

**Methods:**

A sample of 102 obese adolescents (mean age = 15.32 ±1.71) in residential treatment was matched with 102 normal-weight youngsters from the Health Behavior in School-aged Children (HBSC) study (mean age = 15.30 ±1.73).

**Results:**

Adolescents with obesity were significantly more often cyber-victimized than normal-weight peers. Obese youth victimized by traditional bullying experienced lower quality of life, lower motivation for physical activity and higher avoidance and emotional coping towards healthy lifestyles than those non-victimized. Obese cyber-victims experienced significantly higher suicidal ideation.

**Conclusions:**

Traditional and cyber-victimization may hinder treatment effectiveness and healthy lifestyle change in adolescents with obesity. Health professionals should pro-actively address peer victimization and psychosocial functioning during multidisciplinary obesity treatment. Schools could contribute to a better physical and psychosocial health of obese youth by implementing multi-behavioral health-promotion programs.

## Background

Adolescent obesity is increasingly prevalent. The International Association for the Study of Obesity (IASO) and International Obesity Task Force (IOTF) report that 40–50 million school-aged children globally are classified as obese [1]. Adolescent obesity has been linked to various physical health problems and tends to persist into adulthood [2-4]. Besides physical health problems, many adolescents with obesity also experience psychosocial difficulties, such as low self-esteem and depression [5-7].

Treatment programs for children and adolescents with obesity mainly target weight-loss by changing dietary habits, physical activity and parental support [8]. These medical treatment programs are usually supplemented with a lifestyle skills training for a longer sustained effect. In spite of this, their long-term effectiveness has nonetheless been disappointing [8]. Some studies have raised poor psychosocial functioning as a potential barrier to implement these weight-loss interventions and achieve lifestyle change [9-12]. In examining psychosocial functioning of adolescents, not only individual psychopathology but also the context of peer relationships is important. In this context, peer victimization can be a vital contributor to psychosocial malfunctioning [9]. Peer victimization refers to feeling subjected to aggressive acts by other children and is often used synonymously to being bullied, a form of repeated victimization that assumes a power difference between victim and bully [13]. Bullying can be relational, physical, verbal such as teasing, or sexual. It can use traditional face-to-face contact, referred to as traditional bullying, or can employ online communication, referred to as cyberbullying.

Indeed, numerous studies have shown that obese adolescents are especially prone to being traditionally bullied and that this victimization predicts psychosocial difficulties, such as low self-esteem, high depressive symptoms and suicidal ideation [7,14-19]. Their appearance, which is considered a sign of laziness and self-indulgence, serves as a justification to bullies in victimizing obese peers [20]. Adolescents are highly sensitive to being victimized based on their weight since an important part of identity development takes place during adolescence and is intertwined with body image and self-esteem [21]. Further negative effects of peer victimization for obese adolescents are observed in reduced sports enjoyment and motivation for physical activity (PA) [22,23].

Previous research makes a distinction between weight-based teasing during physical activity and outside of physical activity classes. When weight-based teasing occurred during PA, youngsters experienced lower levels of sports enjoyment and of physical activity behaviour. This relation was lessened if the youngsters possessed good problem-solving or avoidant coping skills [22]. When weight-based teasing took place outside of physical activity, it had the opposite effect and seemed to motivate children to increase their exercise levels [22]. Three different mechanisms have been mentioned as possible reasons for lower PA motivation among obese youth. Firstly, avoiding PA has been considered as a coping mechanism to avoid weight-related victimization. Secondly, lower PA motivation may be attributed to poor social reinforcement experiences for physical activity in the past and thirdly, it may be a result of anhedonic feelings and loneliness resulting from victimization [23].

In sum, obese youth are at increased risk for peer victimization, which heightens their risk of psychosocial problems and physical activity avoidance, and thus lowers the effectiveness of professional and lifestyle weight-loss initiatives.

The recent spreading use of digital communication has given rise to a new form of bullying: cyberbullying. In cyberbullying, bullies intentionally and -often but not necessarily- repeatedly send electronic messages with hurtful content, aiming to cause harm or embarrassment to the victim [24]. Prevalence figures of cyberbullying among youngsters range from 3% to 40%, depending on the definition and measure that is used [24,25]. The psychosocial impact of being cyberbullied in a general adolescent population is even more devastating than that of bullying through traditional, face-to-face forms of communication [26].

Adolescents with obesity exhibit more important risk factors for cyber-victimization, such as traditional peer victimization [17,27] and high computer or internet use [28], than normal-weight youth. Cyber-victimization can therefore be expected to be highly prevalent among obese adolescents and to cause more psychosocial problems than traditional bullying.

So far, little is known on cyber-victimization among obese adolescents. Cyber-victimization was examined in the US Health Behaviour in School-Aged Children (HBSC) study in 2005–2006 [29]. They found no association between cyber-victimization and weight group. In total, 9.9% of the teenagers was cyberbullied, this was 11.8% among obese teens. A more recent study in the US investigated cyber-victimization among adolescents attending a weight-loss program [30]. They reported very high cybervictimization rates among those ever victimized (59% to 61% of those ever victimized were cyber-victimized in the past year), but when estimating the prevalence in the total surveyed sample, cyberbullying represented only between 10 to 12%, similar to the rates found in the US HBSC survey. They did not compare these results with a general population sample and therefore do not provide further insight in cyberbullying prevalence rates among obese youth compared to adolescents who have not experienced weight problems. No data is yet available for cybervictimization experiences among obese youth in other countries than the United States. Cross-national differences may however exist in how cyberbullying is experienced as noted in a recent European cross-national study [31].

No study so far examined the association between cyber-victimization among obese youth on the one hand and psychosocial malfunctioning and lower sports motivation as barriers to weight-loss effectiveness on the other hand, despite indications that the psychosocial impact of cyber-victimization can be more extensive than that of traditional victimization. In line with the abovementioned results, cybervictimization could be assumed to have a positive effect rather than a negative one on physical activity motivation, since this form of victimization takes place outside of physical education classes. To the best of our knowledge, no study has yet examined the relation between cybervictimization and physical activity motivation. As physical activity is a key component in obesity treatment, understanding whether traditional and cybervictimization pose risks to the effectiveness of weight-control efforts of obese youth are of great importance.

This current study thus aims to investigate the occurrence of traditional and cyber-victimization among obese adolescents and their association with psychosocial functioning and barriers to lifestyle change.

Research questions addressed in this study were: 1) to what extent are obese adolescents victimized by traditional and cyber-aggression, compared to normal-weight adolescents?; 2) to what extent do obese victims of (cyber-) aggression experience psychosocial problems, compared to non-victims?; and 3) is being (cyber-) aggressed related to more barriers and less facilitators for PA and a healthy diet among obese youth, compared to non-victims? We hypothesized that 1) victimization will be higher among obese youth than among normal-weight peers; 2) victimization and especially cyber-victimization will be positively associated with psychosocial problems; and 3) (cyber-)victims will experience more barriers and less facilitators to PA and a healthy diet than non-victims.

Our study findings could help optimize healthy lifestyle interventions and treatment efforts for obese adolescents by a better understanding of barriers to their effectiveness.

## Methods

### Participants and procedures

Participants were recruited from a residential treatment facility for severe obesity in Flanders, Belgium, to which they were admitted two weeks prior to data-collection (MPC Zeepreventorium De Haan). This facility is the only residential facility in Flanders for obesity treatment and offers a multidisciplinary treatment to severe obesity for a maximum duration of 1 year, after which voluntary outpatient treatment is offered for a maximum of 3 years. Patients obtain their education during their stay at the facility’s school.

All patients (> = 11.5 years old) treated at the time of data-collection (n = 113) and their parents received oral information on the study, after which informed consent was obtained. Patients who were still attending primary school, or for whom either patient or parental informed consent was not available (n = 4), were excluded. Parental consent was obtained on-site during a required information session for parents on obesity treatment, organized by the facility as part of their treatment protocol. This happened one week prior to patient data-collection. All patient data-collection took place in the patient’s small age-related treatment groups (size ca. 20 youngsters). The researcher gave a 5 min introduction to the survey after which participants independently filled out a questionnaire, which took on average 15 min. The study obtained approval from the Ethics Board of the Ghent University Hospital.

The patient data were matched with a sample of 102 normal-weight youngsters drawn from 2 213 normal-weight youngsters in the Flemish HBSC survey (2010). Matching was performed on an individual level, based on gender, age and educational type as these factors can affect traditional or cyber-victimization [24,32]. For grade 7–8, education in Flanders is not fully differentiated in types and this information completely lacked from the HBSC data. For 7^th^-8^th^ graders, Family Affluence (FAS) was used as a proxy, since Flemish HBSC data noted a strong overlap between educational type and Family Affluence [33]. Perfect matches were found for all but one respondent (who was matched with someone from medium FAS instead of low FAS). Family Affluence data was also used for further refined matching in grades 9–12, since both obesity and peer victimization can be influenced by socio-economic status [34,35]. All cases were perfectly matched on age, gender and educational type from age 13.5 on.

### Measures

#### BMI and obesity classification

Height and weight measurement at treatment intake was used to calculate Body Mass Index (BMI). BMI categories were defined using WHO 2007 age and gender-specific cut-offs [36,37]. BMI z-scores were calculated in Epi Info 7.

#### Family affluence

Family Affluence Scale (FAS) was developed by the HBSC study group to overcome the problem of inaccurate adolescent reports of parental occupation or income. It consists of 4 items (i.c. own bedroom, number of holidays per year, number of cars and computers owned) and is a validated self-report measure for adolescents of family wealth [38]. The four items are summed and an index ranging from 0–9 was created. The following, international, cut-off points were used: score of 0, 1, 2 classified as low FAS; score of 3, 4, 5 as medium FAS; score of 6, 7, 8, 9 classified as high FAS.

#### Experiences of bullying

Bullying questions originate from the Flemish HBSC 2010 study. Youngsters indicate their involvement in the past 6 months as bully or victim in traditional bullying (‘bullied at school’) or as victim in cyberbullying (‘via Internet, e.g. chat or e-mail, or via mobile phone, e.g. text messages’). The cyberbully role was only questioned in the group of obese adolescents. Bullying questions are introduced by a description that differentiates bullying from quarrels or disagreements between friends. The questions are answered on a 5-point Likert scale ranging from ‘never in the past 6 months’ to ‘several times per week’. This method commonly used in HBSC studies has been previously described elsewhere [39]. Additionally in the group of obese adolescents, youngsters were asked about the location where traditional bullying took place (physical education class, regular class, the playground, to and from school), and about specific forms of cyberbullying they had experienced (receiving personal hurtful messages, rumours or photos spread to others, vicious acts such as profile hacking, being socially excluded). These more detailed questions on victimization showed high overlap with the single item measured in the HBSC survey, lending support to using this single item to measure peer victimization.

For logistic regression analysis and comparisons by respondent characteristics, bullying involvement was dichotomized into ‘never’ and ‘at least once in past 6 months’. This transformation was needed since data for the bullying questions was severely skewed by a low involvement in bullying. Numbers in each cell would hence have been too small to allow statistical comparisons using the more rigorous definition posited by Olweus, according to which bullying needs to happen repeatedly (i.c. at least 2–3 times per month) to qualify as victimization. As repetitiveness of victimization is considered a key aspect of bullying, the dichotomous measure we used here is not referred to in this paper as cyberbullying or traditional bullying, but as cyber aggression and traditional aggression. The continuous measure of victimization implying repetitiveness, is only used in the first part of 3.2, where it is referred to as cyberbullying or traditional bullying.

Bullying and aggression perpetration are briefly mentioned in part 3.2 but not used for further comparisons or predictions, as this study focuses on victimization experiences.

#### Quality of life

The KIDSCREEN-10 was used to assess quality of life. The KIDSCREEN-10 consists of ten items (e.g. ‘Have you felt sad?’; ‘Have you been able to do the things that you want to do in your free time?’) which are answered on a 1–5 Likert scale. Two items (school performance, physical fitness) were omitted since our survey was administered during the start of school holidays and as physical fitness is naturally lower among adolescents with obesity entering treatment. The KIDSCREEN-10 instrument is a valid and reliable measure of quality of life and contains indications of general energy level, depressive emotions, leisure time enjoyment, relationship with parents and peers, and perception of cognitive capacity [40]. The eight items composing the KIDSCREEN measure were averaged into one single quality-of-life index. Responses were recorded so that higher values represented better quality of life. Internal consistency of the KIDSCREEN with 8 items in the sample of patients with obesity was sufficient (Cronbach’s α =0.77). For logistic regression analysis, the quality of life index was dichotomized via the median.

#### Suicidal ideation

Suicidal ideation was measured by a single item originating from the Flemish HBSC survey: ‘Have you ever thought about ending your life?’, answered on a 5-point scale ranging from ‘never’ to ‘very often’. For logistic regression analysis, the suicidal thoughts question was dichotomized into ‘never’ and ‘at least once’.

#### Positive global self-esteem

Positive global self-esteem was measured by a single item from the Rosenberg Self-Esteem Scale (RES), namely ‘I take a positive attitude toward myself’. Global self-esteem can be measured by a single item [41] and this specific item is a main contributor to global positive self-esteem [42,43]. For logistic regression analysis, the self-esteem item was dichotomized via the median.

#### Barriers and facilitators to physical activity and healthy diet after peer victimization

Literature documents several coping behaviours which alter the impact of weight-based victimization on healthy lifestyles [10,22,23,44-46]. Nine items reflecting re-occurring themes were constructed after reviewing this literature (see Table 1). Items were answered on a 5-point Likert scale ranging from ‘not at all applicable to me’ to ‘completely applicable to me’. To the best of our knowledge, no validated scales exist to measure this. Faith and colleagues [22] adapted a 30-item coping scale to assess coping with weight criticism. As our purpose is to examine a wider concept of victimization than weight criticism and given the concern with questionnaire length, we chose to construct a new shorter scale that closer fits our research questions. Content validity was established in discussion with prominent clinicians and researchers in obesity treatment. Principal component analysis with Varimax rotation was conducted to find homogeneous concepts. After initial analysis, the item ‘being slim as a goal’ was dropped from analysis because of low contribution to internal consistency of the factor. Three factors explained 66% of the variance in the remaining eight items. A first factor was named avoidance and emotional coping (Cronbach’s α = .70), a second factor referred to problem-solving coping (Cronbach’s α = .69) and a last factor reflected intrinsic motivation for PA (Cronbach’s α = .46). Table 1 shows descriptives for these factors. The three factors on barriers and facilitators were calculated by taking the mean score of the items belonging to each factor.

**Table 1:** Factor structure of barrier and facilitator scale

	**M**	**SD**	**Loading**
***Factor 1: Avoidance and emotional coping***			
I avoided sports because I was afraid they would laugh at me	2.35	1.38	.830
When I felt lonely or sad, I sometimes ate to feel better	2.54	1.40	.789
I avoided socializing with peers who are slimmer than me	1.63	0.88	.691
***Factor 2: Problem-solving coping***			
When I was criticized for not being sufficiently sportive, I tried to do something about it	2.69	1.13	.798
I only ate fruit, vegetables and other low-calorie-food	1.90	0.97	.773
I minded my weight to be liked by others	2.50	1.13	.738
***Factor 3: Motivation for physical activity***			
Being fit and sportive were important goals for me	3.33	1.24	.822
I enjoyed sports and exercise	3.20	1.15	.699

### Statistical analysis

Statistical analyses were conducted using SPSS (version 21). As mentioned above, some questions were transformed before analysis. Chi square tests were used to study the significance of differences in bullying involvement between obese and non-obese youth. Logistic regression analysis was used to address the main research questions, since many items were not normally distributed and loglinear transformation did not solve this problem. Victimization was categorized using the dichotomization described above. Independent variables controlled for in the analysis were age, gender (boys, girls) and family affluence (low and medium, high). For all research questions, age, gender and family affluence were added as covariates in a multivariate logistic regression. For research question 2 and 3, victimization of one form was also added as a covariate to the predictive value of the other form of victimization. As there were very few missing values to the variables used in the analyses, respondents with missing information on a specific variable were considered as a missing case for that particular analysis.

## Results

### Descriptive data

One parent and three adolescents did not provide informed consent. These four patients were excluded from participating. One-hundred and nine obese adolescents filled out the questionnaire. From this sample, seven children were removed since they still attended primary school and are considered pre-adolescents.

In total, 102 obese adolescents participated. They were matched with 102 normal-weight peers. Sample characteristics are provided in Table 2.

**Table 2:** Sample characteristics

	**Obese adolescents**	**Normal-weight adolescents**
	**(n = 102)**	**(n = 102)**
**Gender**		
Male	n = 39 (38.2%)	n = 39 (38.2%)
Female	n = 63 (61.8%)	n = 63 (61.8%)
**Age**		
Average (±SD)	M = 15.32 ±1.71	15.30 ±1.73
11-12y	n = 4 (3.9%)	n = 4 (3.9%)
13-14y	n = 29 (28.4%)	n = 29 (28.4%)
15-16y	n = 43 (42.2%)	n = 43 (42.2%)
17-18y	n = 26 (25.5%)	n = 26 (25.5%)
Education*		
General	n = 24 (30.0%)	n = 24 (30.0%)
Technical or vocational	n = 56 (70.0%)	n = 56 (70.0%)
*Missing*	n = 4	n = 4
**Family affluence**		
Low (0–2)	n = 4 (3.9%)	n = 0 (0.0%)
Medium (3–5)	n = 39 (38.6%)	n = 37 (36.3%)
High (6–9)	n = 58 (57.4%)	n = 65 (63.7%)
*Missing*	n = 1	n = 0
**Country of birth**		
Belgium	n = 95 (93.1%)	n = 91 (89.2%)
Other country	n = 7 (6.9%)	n = 11 (10.8%)
*Missing*	n = 0	n = 0
**Weight status**		
Average BMI (±SD)	37.86 ±5.97	21.43 ±1.65
Average BMI z-score (±SD)	3.46 ±0.81	0.46 ±0.33

*figures only available from grade 9 onwards.

### Peer victimization in obese and non-obese youth

This part firstly describes the extent to which traditional and cyber-victimization occurs in obese and non-obese youth, next compares whether these rates differ between obese and non-obese adolescents, and lastly, characteristics of obese and non-obese victims are described.

The majority of adolescents with obesity was not bullied in the past 6 months, either via traditional or electronic means. The rate of traditional victimization was higher than for cyber-victimization. Following Olweus’ definition of bullying involvement (i.e. at least two to three times per month), 5.9% (n = 6) of non-obese adolescents were traditional victims, 6.0% (n = 6) were traditional bullies and 2.9% (n = 3) were cyber-victims (no info available on cyber-bullies). Among the obese adolescents, 12.9% (n = 13) were traditional victims, 8.0% (n = 8) were traditional bullies, 4.0% (n = 4) were cyber-victims, 4.0% (n = 4) were cyber-bullies. Most common location for being bullied among obese adolescents was the playground (n = 13, 12.7%), followed by regular class (n = 11, 10.8%), during physical education class (n = 9, 8.8%) and lastly to and from school (n = 8, 7.8%). The most prevalent form of cyber-bullying among obese adolescents was social exclusion (n = 7, 6.8%), followed by rumours or embarrassing images sent to others (n = 6, 5.9%), receiving hurtful messages (n = 3, 2.9%) and being hacked or intentionally receiving a virus (n = 1, 1.0%).

Because of low victimization experience we will deviate from Olweus’ definition for further analyses and instead consider any victimization/perpetration in the past 6 months as victim/bully. This approach has been used in prior cyberbullying studies [29,39].

According to this last definition, there are twice as many obese adolescents (n = 17, 17.2%) as normal-weight adolescents (n = 8, 7.8%) who are cyber-victims, which is statistically significant (χ^2^(1) = 4.014, p < .05). There are also more obese victims of traditional aggression (n = 32, 31.7%) than normal-weight youth (n = 22, 21.8%), but this difference does not reach significance (χ^2^(1) = 2.024, p = ns). There are no significant differences (χ^2^(1) = 0.570, p = ns) between obese adolescents (n = 30, 30.0%) and normal-weight adolescents (n = 35, 35.0%) in involvement as perpetrator in traditional aggression. No comparison is available for cyber-perpetrators.

There is a strong overlap in victimization among obese youth from traditional aggression and cyber aggression: 76.5% (13/17) of the cyber-victims among obese youth are also traditional victims. Traditional victims consequently are also 17 times more likely to be cybervictims.

Table 3 shows that obese youth are more than twice as likely to be the victim of cyber aggression compared to normal-weight youth, controlling for age, gender and family affluence as covariates. The odds to be victimized by traditional aggression was not significantly higher for obese youth (odds ratio = 1.746) than for normal-weight youth.

**Table 3:** Prediction of traditional victimization and cyber-victimization by weight status, controlled for demographic variables

	**Odds ratio (95% CI)**	**Adjusted odds ratio (95% CI)**
***Dependent: traditional victimization***		
	*Model 1*	*Model 2*
Obesity^a^	1.665 (0.886-3.132)	1.744 (0.915-3.323)°
Age		0.784 (0.645-0.952)*
Gender (girl)^b^		1.039 (0.538-2.005)
Family Affluence (high)^c^		0.950 (0.494-1.829)
***Dependent: cybervictimization***		
	*Model 3*	*Model 4*
Obesity^a^	2.436 (0.999-5.938)°	2.547 (1.017-6.379)*
Age		0.647 (0.486-0.861)**
Gender (girl)^b^		1.176 (0.474-2.916)
Family Affluence (high)^c^		0.702 (0.292-1.688)

*°p* <.1*;* *p < .05; **p < .01.

Reference category = 0; ^a^0 = normal-weight youth, 1 = obese youth; ^b^0 = boy, 1 = girl; ^c^0 = low and medium FAS, 1 = high FAS.

Model 1: -2LL = 232.016; Nagelkerke R^2^ = 0.018; Model χ^2^(1) = 2.539, p = 0.111.

Model 2: -2LL = 224.849; Nagelkerke R^2^ = 0.064; Model χ^2^(4) = 9.082; p = 0.059.

Model 3: -2LL = 146.886; Nagelkerke R^2^ = 0.038; Model χ^2^(1) = 4.088; p = 0.043.

Model 4: -2LL = 135.898; Nagelkerke R^2^ = 0.137; Model χ^2^(4) = 15.076; p = 0.005.

### Psychosocial problems related to (cyber-) aggression among obese youth

The following results only relate to obese youth. Given the strong overlap in victimization from cyber aggression and traditional aggression, we could not distinguish pure cyber-victims from pure traditional victims or from victims of both forms. The results discussed here are taking the covariates of traditional/cyber-victimization, age, gender and family affluence into account, as shown in Table 4.

**Table 4:** Prediction of psychosocial distress by traditional victimization and cyber-victimization among obese youth, controlled for demographic variables

	**Odds ratio OR (95% CI)**		
	**Self-esteem**	**Quality of life**	**Suicidal thoughts**
Unadjusted OR			
	*Model 1*	*Model 2*	*Model 3*
Traditional victimization (victim)^a^	0.529 (0.215-1.297)	0.342 (0.144-0.812)*	3.533 (1.438-8.685)**
Unadjusted OR			
	*Model 4*	*Model 5*	*Model 6*
Cybervictimization (victim)^b^	0.595 (0.199-1.780)	0.404 (0.139-1.172)°	6.315 (2.051-19.444)**
Adjusted OR			
	*Model 7*	*Model 8*	*Model 9*
Traditional victimization (victim)^a^	0.583 (0.205-1.662)	0.325 (0.115-0.916)*	2.421 (0.829-7.067)
Cybervictimization (victim)^b^	0.589 (0.162-2.138)	0.502 (0.141-1.788)	5.647 (1.534-20.787)**
Age	0.836 (0.635-1.101)	0.798 (0.611-1.043)°	1.217 (0.909-1.629)
Gender (girl)^c^	0.628 (0.242-1.629)	0.430 (0.169-1.094)°	1.304 (0.474-3.583)
Family Affluence (high)^d^	0.733 (0.293-1.836)	0.748 (0.306-1.826)	1.465 (0.541-3.970)

°*p* < .1; *p < .05; **p < .01.

Reference category = 0; ^a^0 = not victimized by traditional aggression, 1 = victimized by traditional aggression; ^b^0 = not victimized by cyber aggression, 1 = victimized by cyber aggression; ^c^0 = boy, 1 = girl; ^d^0 = low and medium FAS, 1 = high FAS.

Model 1: -2LL = 120.286; Nagelkerke R^2^ = 0.028; Model χ^2^(1) = 1.925, p = 0.165.

Model 2: -2LL = 131.069; Nagelkerke R^2^ = 0.079; Model χ^2^(1) = 6.072; p = 0.079.

Model 3: -2LL = 116.122; Nagelkerke R^2^ = 0.104; Model χ^2^(1) = 7.698; p = 0.006.

Model 4: -2LL = 120.549; Nagelkerke R^2^ = 0.012; Model χ^2^(1) = 0.846; p = 0.358.

Model 5: -2LL = 130.736; Nagelkerke R^2^ = 0.038; Model χ^2^(1) = 2.838; p = 0.092.

Model 6: -2LL = 107.381; Nagelkerke R^2^ = 0.152; Model χ^2^(1) = 10.957; p = 0.001.

Model 7: -2LL = 114.277; Nagelkerke R^2^ = 0.071; Model χ^2^(5) = 4.920; p = 0.426.

Model 8: -2LL = 119.419; Nagelkerke R^2^ = 0.169; Model χ^2^(5) = 13.113; p = 0.022.

Model 9: -2LL = 102.672; Nagelkerke R^2^ = 0.212; Model χ^2^(5) = 15.665; p = 0.008.

Obese traditional victims were three times more likely (inverted odds ratio = 1/0.325) to have a low quality of life than obese non-victims of traditional aggression. The odds for a low self-esteem and ever having had suicidal thoughts were not significantly different between obese victims and non-victims of traditional aggression. For obese cyber-victims, the odds of having a low quality of life or low self-esteem did not differ significantly from obese youth not cyber-victimized. Obese cyber-victims were however more than 5 times as likely to have ever thought about committing suicide compared to obese non-victims of cyber aggression.

Information for normal-weight youth is only available for quality of life and for suicidal thoughts. As for obese youth, normal-weight youth who are cybervictimized are 5 times as likely to ever have thought about committing suicide compared to normal-weight non-victims of cyber aggression. Normal-weight youth who are traditional victims have however no significantly lower quality of life than those non-victimized, while for obese youth, traditional victims did experience a lower quality of life than their non-victimized peers.

### Perceived barriers and facilitators towards PA and a healthy diet among obese youth

Obese traditional victims were 3.6 times more likely to have a high use of avoidance and emotional coping than non-victims (see Table 5). They were also less likely to be intrinsically motivated for physical activity: traditional victims were 4.6 times (inverted odds ratio = 1/0.217) more likely to have a low enjoyment and low motivation for sports than those who were not victimized by traditional aggression. There were no significant differences in odds to use problem-solving strategies between victims and non-victims of traditional aggression.

**Table 5:** Prediction of healthy lifestyle barriers and facilitators by traditional victimization and cybervictimization among obese youth, controlled for demographic variables

	**Odds ratio OR (95% CI)**		
	**Avoidance and emotional coping**	**Problem-solving coping**	**Motivation for physical activity**
Unadjusted OR			
	*Model 1*	*Model 2*	*Model 3*
Traditional victimization (victim)^a^	3.007 (1.244-7.266)*	1.919 (0.718-5.132)	0.388 (0.160-0.946)*
**Unadjusted OR**			
	*Model 4*	*Model 5*	*Model 6*
Cybervictimization (victim)^b^	1.815 (0.616-5.350)	1.313 (0.391-4.408)	0.858 (0.292-2.522)
**Adjusted OR**			
	*Model 7*	*Model 8*	*Model 9*
Traditional victimization (victim)^a^	3.620 (1.291-10.151)*	2.001 (0.683-5.865)	0.217 (0.069-0.677)**
Cybervictimization (victim)^b^	1.160 (0.326-4.129)	0.850 (0.649-1.114)	0.785 (0.601-1.027)
Age	1.215 (0.939-1.573)	0.722 (0.289-1.807)	1.105 (0.457-2.672)
Gender (girl)^c^	1.485 (0.609-3.617)	1.443 (0.562-3.705)	0.356 (0.142-0.892)
Family Affluence (high)^d^	1.141 (0.479-2.718)	0.805 (0.205-3.158)	1.448 (0.379-5.528)

°*p* < .1; *p < .05; **p < .01.

Reference category = 0; ^a^0 = not victimized by traditional aggression, 1 = victimized by traditional aggression; ^b^0 = not victimized by cyber aggression, 1 = victimized by cyber aggression; ^c^0 = boy, 1 = girl; ^d^0 = low and medium FAS, 1 = high FAS.

Model 1: -2LL = 131.747; Nagelkerke R^2^ = 0.081; Model χ^2^(1) = 6.242, p = 0.012.

Model 2: -2LL = 112.982; Nagelkerke R^2^ = 0.028; Model χ^2^(1) = 1.746; p = 0.186.

Model 3: -2LL = 133.929; Nagelkerke R^2^ = 0.059; Model χ^2^(1) = 4.541; p = 0.033.

Model 4: -2LL = 133.204; Nagelkerke R^2^ = 0.016; Model χ^2^(1) = 1.180; p = 0.277.

Model 5: -2LL = 115.059; Nagelkerke R^2^ = 0.003; Model χ^2^(1) = 0.197; p = 0.658.

Model 6: -2LL = 135.411; Nagelkerke R^2^ = 0.001; Model χ^2^(1) = 0.078; p = 0.780.

Model 7: -2LL = 123.605; Nagelkerke R^2^ = 0.126; Model χ^2^(5) = 9.616; p = 0.087.

Model 8: -2LL = 109.315; Nagelkerke R^2^ = 0.067; Model χ^2^(5) = 4.286; p = 0.509.

Model 9: -2LL = 119.773; Nagelkerke R^2^ = 0.185; Model χ^2^(5) = 14.440; p = 0.013.

Obese cyber-victims did not have significantly different odds in any of the facilitators or barriers towards physical activity and a healthy diet compared to non-cybervictims.

These questions were not available in the HBSC study and hence no comparison with non-obese youth can be made.

## Discussion

This study investigated the extent of traditional and cyber-victimization among obese youth, and its relation to psychosocial factors and to barriers for a healthy diet and physical activity.

### Victimization rates

Our study showed that compared to normal-weight youngsters, significantly more obese adolescents had experienced cybervictimization at least once in the past six months (7.8% versus 17.2% respectively). Literature to compare our results with on cyber-victimization among obese youth is still very scarce. Puhl and colleagues [30] used a US clinical sample of overweight, obese and previously overweight/obese adolescents. Obese youth did not differ in odds of cybervictimization from those who were previously overweight/obese. The study however concluded that also youth who were previously obese were still highly victimized and that this comparison group does not truly represent normal-weight youngsters in a general population. Further comparisons are also hampered by a deviating bullying operationalization from what is commonly used [24]. A second study on cyberbullying among obese youth was conducted on US HBSC data [29]. They found no significant differences between youth with or without obesity in cybervictimization. Several sample dissimilarities could underlie this discrepancy. Firstly, our study used a clinical sample while the US HBSC study was conducted in the general community. Treatment-seekers are usually more troubled than obese adolescents in the general population [7], which may explain our higher occurrence of cybervictimization since having socio-emotional and psychological problems is a risk factor for peer victimization [47,48]. Secondly, our sample consisted of youth with severe obesity with a potentially higher BMI than the US HBSC obese sample. Obese community groups often have significantly lower BMI than clinical groups [7]. Furthermore, very muscular adolescents who draw less stigmatizing attention can be misclassified as obese in community samples [49]. These are not included in our clinical sample of adolescents screened for obesity by health professionals. Based on these assumptions, extreme obesity may be needed as a threshold to elicit cybervictimization, similarly to what has been documented for traditional bullying, but at a much higher cut-off point. And lastly, as obesity is more common in US culture than in Belgium [50], US obese youth may be less stigmatized. Weight stigmatization has however shown not to decrease as obesity becomes more prevalent [51]. It is however possible that cybervictimization is not experienced in the same way in different countries [31]. More cross-national studies are needed to further shed light on these variations. In sum, our finding that obese youth is more often cyber-victimized is not consistent with the scarce literature on this topic, possibly due to sample or measurement differences.

Our results further show that for both obese and normal-weight youngsters, victimization from traditional aggression was more prevalent than from cyber aggression. More obese adolescents (32%) than normal-weight adolescents (23%) had been the victim of traditional aggression, but this was not significant. Most studies on weight-based victimization have reported victimization rates for obese youth up to twice as high as for youth who are not obese [17]. A recent study found a greatly but non-significantly higher risk for peer victimization among obese youth, due to largely varying odds ratio’s [18]. This is consistent with our findings. Our study results also show that obese youth have on average a higher risk to be traditional victims, but that this increased risk is not statistically significant because of large variations in this risk among the studied youth. Some other personal or environmental factors may be at play here which make one adolescent with obesity at much higher risk to be traditionally victimized and others not. Otherwise put, this large variation in odds ratios could point to important mediating and moderating factors in risk of peer victimization. One such mediating or moderating factor could be self-esteem [19,52,53], which may be important to integrate in programs to avoid victimization in this at-risk group.

### Psychosocial functioning

Being a traditional victim among our obese participants was significantly related to a lower quality of life. This is consistent with earlier studies on the correlates of peer victimization among obese youth [19,21,52,53] and on those of weight-based teasing [15,16]. Normal-weight youth who were traditional victims did not experience a lower quality of life and thus seem less affected by traditional aggression than obese youth.

Traditional peer victimization among obese youth was not found to be related to a lower self-esteem or to ever having had suicidal thoughts. Peer victimization is but one factor that could contribute to low self-esteem and suicidal thoughts besides other important risk factors such as body image [54], or psychopathological risk factors, such as depression or substance abuse [55], which were not included in our study. Obese adolescents with a positive body image have a higher self-esteem and high well-being [7]. As our study did not include an indication of body image, we cannot determine if a more positive body image protected against the negative effects of peer victimization on self-esteem. Being in residential care among obese peers may have caused participants to feel better about their own appearance. In another study on obese youth in residential care, body dissatisfaction did decrease after time in treatment [56]. Although our study was conducted immediately after admission to the facility, being among other obese peers may have still impacted their body image positively.

Being a cyber-victim was unexpectedly not significantly related to a lower quality of life and lower self-esteem among the obese adolescents in our study. Previous general adolescent population studies showed cyberbullying to have a greater psychosocial impact than traditional bullying [26,57]. This was not found in our obese sample in spite of the type of cyberbullying considered most harmful, i.c. spreading rumours and photo’s [58], being quite common among our participants. Some research suggested that for pure cyber-victims, this victimization experience has a negative association with their self-esteem. However, when a person is both cyberbullied and traditionally bullied, the additional impact of cyber-victimization is negligible [25]. As the large majority of our patients with obesity who were victimized experienced both victimization forms, we could not assess the psychosocial impact for pure cybervictims. Another possible explanation is that our obese youth who were cyber-victimized were not as frequently bullied as those victimized via traditional bullying, while repetition of peer victimization is nevertheless an important factor in how much psychosocial harm it is linked to [25].

Cyber-victimization was nonetheless significantly related to having considered suicide at least once. The association between peer victimization and suicidal ideation has been extensively demonstrated before [55,58,59]. Depression is often a mediating factor, but direct effects of peer victimization on suicidal ideation do remain [59,60]. Furthermore, victimization does not require repetition to increase the risk of suicidal ideation, unlike for other psychosocial harm [61]. Suicidal ideation is a complex process, with distal and proximal risk factors which precipitate plans into action. Stressful life events, such as peer victimization, fall under the latter category and are considered neither necessary nor sufficient to translate suicidal thoughts into committing suicide [62]. Our suicidal ideation measure was dichotomized into ‘never’ and ‘ever’ for a better fit with the data. The latter category does however not necessarily imply current suicidal ideation, since past suicidal ideation has shown not to significantly predict later suicidal ideation at a long-term follow-up [63]. But given that the rate of making suicide plans is similar between youngsters who consider suicide only once and those who experience persistent suicidal ideation, even one-time suicidal ideation does form a cause of concern [64]. Therapists treating patients when they committed suicide also point to the importance of acute intense emotions, such as humiliation, as contributors to suicide risk [65]. Especially for cyberbullying, the purpose is more public humiliation than asserting power over the victim. Possibly cyber-victimization triggers suicidal ideation to escape humiliation, while victimization from traditional bullying aimed at rendering the victim powerless would be stronger associated with depressive symptoms. By only screening for psychopathology in suicide risk assessment, these acute suicidal reactions to humiliation after cyber-victimization, also featuring in recent media reports, would be missed. This hypothesis clearly warrants further research.

Summarized, both traditional and cyber-victimization were related to different aspects of psychosocial distress among obese youth, with three- to-five fold increased odds of psychosocial harm compared to non-victims. Traditional victims who were obese were more affected than normal-weight traditional victims.

### Barriers and facilitators to healthy lifestyles

Our findings indicated that neither traditional nor cyber-victimization was related to problem-solving coping in physical activity (PA) and healthy eating. One other study suggested positive, problem-solving reactions to weight criticism, which would then lead to greater PA among adolescents [22]. This was not confirmed in our study. Instead, traditional victimization related to avoidance of PA, of contact with lower-weight peers and to more emotional eating. Furthermore, traditional victimization was also associated with decreased motivation and enjoyment for PA. Based on Self-Determination Theory, intrinsic motivation is the best guarantee to a maintained level of physical activity [66]. Our study indicates that traditional peer victimization seriously hinders the much-needed physical activity for adolescents with obesity to reduce weight. Physical education (PE) teachers play an important role in creating a supportive environment for PA and exercise enjoyment among adolescents [66]. Many PE teachers nevertheless do not appropriately intervene in situations of weight-based victimization, increasing the risk of sports avoidance among those victimized [67]. Cybervictimization was not related to higher sports avoidance. It is possible that because the victimization takes place outside of a exercise setting this has less influence on sports motivation, as found in the study by Faith et al. [22].

This study’s findings were innovative in establishing a link between cyber-victimization and potential psychosocial barriers to weight-loss effectiveness for obese youth. The study also deepened insights on the effects of traditional bullying on avoidance of healthy lifestyles and reduced exercise motivation. Our results indicate that weight-loss interventions for obese adolescents should look beyond mere nutrition and physical activity and include a broader scope on healthy lifestyles and psychosocial functioning, which may hinder or facilitate weight-loss. Health professionals should be mindful to signs of traditional and cyber-victimization and proactively discuss peer relationships, teach problem-solving skills and ensure high self-esteem as part of a multi-component program. School environments are well-accepted settings by both students and staff to support adolescents’ emotional health [68] and a positive school climate and high school connectedness have also been established as protective factors against peer victimization and low emotional well-being [69,70]. Schools should consequently implement an effective approach to restrict peer victimization and improve youngsters’ psychosocial functioning and healthy lifestyle adoption. Multi-behavioural whole-school programs [71] that target a wide variety of healthy lifestyles including physical activity and positive social behaviour, are promising recent developments that could benefit all youngsters, but in particular obese youth who are in high need of healthy lifestyle promotion.

### Limitations and future research

The study had several limitations. First, the study is cross-sectional and causality of the found associations can therefore not be concluded. Second, reliability and validity tests of the scale to measure barriers and facilitators for a healthy lifestyle were limited and future research should establish other psychometric properties of the scale. Third, using a more comprehensive scale for self-esteem may provide a better estimate than the single-item scale used here. Fourth, as this was a clinical sample, the results may not generalize to adolescents with obesity in the general population. Furthermore, our sample was too small to allow differentiation between pure victim roles or to find gender differences among cyber-victims. The low number of victims in the study precluded the analysis of continuous measures and of control variables. As a sample of adolescents with severe obesity, it is however quite substantial. With a low prevalence of cyberbullying in this small sample, it furthermore implied deviating from the definition of bullying stated by Olweus which requires victimization to take place repeatedly. Although this is not an uncommon approach in cyberbullying research, it may have affected the strength of the relationship with psychosocial functioning.

The study’s strengths lie in its matched sample between adolescents with obesity and with normal weight, which filtered out the influence of certain determinants of either obesity or victimization, such as gender, age, and socio-economic status. The dataset from which the matched sample was drawn, was sufficiently large to provide identical matches for all but one respondent. Although HBSC data was collected two years prior to data-collection among youth with obesity, this does not jeopardize comparability since cyber-bullying rates have been relatively stable in recent years [25,72]. Future longitudinal studies could help clarify the direction of the relation between victimization among obese youth and psychosocial distress. The integration of both weight-status measures and quality of life indices is needed in large-scale studies on either cyberbullying or adolescent health to gain more insight in psychosocial harm for pure traditional, pure cyber- or combined victims. Furthermore, having a broader range of weight-statuses among study participants could validate the assumed threshold in obesity that elicits increased cyber-victimization and could enable comparisons in psychosocial harm and barriers to healthy lifestyles by weight status. Future studies should use multiple estimates for psychosocial harm and barriers to healthy lifestyles since different forms of bullying may affect youth differently. As we found differences between US samples and our Belgian study, a cross-country comparison assessing cultural influences may also be of value in future research. And lastly, a qualitative study among obese cyber-victims to explore suicidal ideation could shed light on its mechanisms and provide further suggestions for prevention and clinical care.

## Conclusions

Obese youth were 2.5 times more likely to be the victim of cyber-bullying than non-obese youth. Both victimization from cyber-bullying and from traditional bullying were associated with lower psychosocial health among obese youth, but affected youngsters differently. The recurrent question in bullying research whether psychosocial harm is highest when resulting from traditional victimization or from cybervictimization is perhaps less relevant than the focus on which harm results from which type of victimization. Traditional victims were three times more likely to have a low quality of life, while cyber-victims were 5.6 times more likely to ever having had suicidal thoughts. Cybervictimization may heighten suicidal ideation in absence of an impact on quality of life by an acute reaction of embarrassment. These mechanisms need further investigation in future cyberbullying research. Traditional victimization was furthermore related to a higher avoidance of healthy lifestyles and more emotional coping, and to a lower enjoyment of sports and intrinsic motivation for physical activity. This was not the case for cyberbullying, which may confirm earlier suggestions that weight-based criticism affects physical activity motivation only when it takes place during exercise.

## Abbreviations

BMI: Body mass index; FAS: Family affluence scale; HBSC: Health behavior in school-aged children; OR: Odds ratio; PA: Physical activity; PE: Physical education; US: United States.

## Competing interests

Ann DeSmet is a recipient of a PhD-scholarship from the Flemish agency for Innovation by Science and Technology (B/12754/01). Benedicte Deforche, Anne Hublet, Ann Tanghe, Evi Stremersch and Ilse De Bourdeaudhuij have no financial disclosures. The authors declare no conflict of interests.

## Authors’ contributions

AD, BD, AH, AT, ES and IDB developed the questionnaire and research protocol. AD, AT and ES collected the data among obese youth, AH collected the data among normal-weight youngsters. AD, BD and IDB conducted the data-analysis. AD drafted the manuscript. All authors revised and approved the final manuscript.

## Pre-publication history

The pre-publication history for this paper can be accessed here:

http://www.biomedcentral.com/1471-2458/14/224/prepub
